# Magnetic characterization of cobalt nanowires and square nanorings fabricated by focused electron beam induced deposition

**DOI:** 10.3762/bjnano.9.97

**Published:** 2018-04-03

**Authors:** Federico Venturi, Gian Carlo Gazzadi, Amir H Tavabi, Alberto Rota, Rafal E Dunin-Borkowski, Stefano Frabboni

**Affiliations:** 1 FIM Department, University of Modena and Reggio Emilia, Via G. Campi 213/a, Modena I-41125, Italy; 2 CNR – Nanoscience Institute, S3 Center, Via G. Campi 213/a, Modena I-41125, Italy; 3 Ernst Ruska-Centre for Microscopy and Spectroscopy with Electrons and Peter Grünberg Institute, Forschungszentrum Jülich, 52425 Jülich, Germany; 4 Intermech-Mo.Re. Center, University of Modena and Reggio Emilia, Via Vignolese 905/b, Modena I-41125, Italy

**Keywords:** focused electron beam induced deposition, magnetic force microscopy, magnetic nanostructures, off-axis electron holography, transmission electron microscopy

## Abstract

The magnetic properties of nanowires (NWs) and square nanorings, which were deposited by focused electron beam induced deposition (FEBID) of a Co carbonyl precursor, are studied using off-axis electron holography (EH), Lorentz transmission electron microscopy (L-TEM) and magnetic force microscopy (MFM). EH shows that NWs deposited using beam energies of 5 and 15 keV have the characteristics of magnetic dipoles, with larger magnetic moments observed for NWs deposited at lower energy. L-TEM is used to image magnetic domain walls in NWs and nanorings and their motion as a function of applied magnetic field. The NWs are found to have almost square hysteresis loops, with coercivities of ca. 10 mT. The nanorings show two different magnetization states: for low values of the applied in-plane field (0.02 T) a horseshoe state is observed using L-TEM, while for higher values of the applied in-plane field (0.3 T) an onion state is observed at remanence using L-TEM and MFM. Our results confirm the suitability of FEBID for nanofabrication of magnetic structures and demonstrate the versatility of TEM techniques for the study and manipulation of magnetic domain walls in nanostructures.

## Introduction

Magnetic nanostructures are studied intensively for their applications in high-density data storage [1–2], magnetic random access memory [3], magnetic logic nanodevices [4] and magnetic sensing [5]. A new concept of fast memory, which is referred to as racetrack memory, has been proposed, based on the motion of domain walls along a nanowire (NW) subject to current pulses or external magnetic fields [6]. The strong research interest in such new types of memories is based on their promise for reliable, high-capacity and high-performance devices [7]. However, in all of the proposed applications, the stability of the magnetic state of the nanostructure depends on factors such as its composition, crystal structure and shape [8].

Co-based magnetic nanostructures can be deposited by focused electron beam induced deposition (FEBID) of Co carbonyl (Co_2_(CO)_8_). This is a direct-write technique performed in a scanning electron microscope (SEM) equipped with a gas injector system (GIS) [9]. It exploits secondary electron emission resulting from interaction of the primary electron beam with a substrate to decompose molecules that are adsorbed on the surface. The non-volatile part of the molecule is deposited, whereas volatile ligands are pumped away. FEBID is a versatile technique for nanoprototyping and research, as it permits the deposition of material in a variety of shapes with high spatial resolution. Moreover, it is also possible to deposit oxides [10] and, by co-injecting different precursors, alloy materials with tunable properties [11]. A review of the application of the technique to the deposition of magnetic nanostructures has recently been published [12]. It should be noted that Co carbonyl precursor was chosen here because it has been shown to provide high purity deposits with magnetic properties that are close to those of pure Co [13].

It is well known that shape anisotropy has a profound influence on the magnetic properties of nanostructured materials. The NW is a basic building block of magnetic nanodevices, as its high aspect ratio (length/width) often results in a single magnetic domain state due to shape anisotropy [14]. In detail, a NW has a stable magnetic state if its width is smaller than 7·Δ_d_, where Δ_d_ is the dipolar exchange length [15], which is ca. 3.4 nm for Co [16]. Ring shapes have also been proposed as elements for magnetic memories thanks to their higher stability compared to filled shapes [17]. Square nanorings, in particular, are attractive since their right-angle vertices provide well-defined reference points for magnetization orientation, while magnetostatic interactions between different sides give rise to different possible magnetization states [18]. Extensive work has previously been performed on Co nanostructures using magnetic force microscopy (MFM) [19–20], Lorentz-transmission electron microscopy (L-TEM) [21] and electron holography (EH) [22].

Here, we use different techniques and different magnetization conditions to investigate the magnetic states of NWs and square nanorings formed from four NWs. The magnetization states of the square nanorings are studied both in the presence of an applied magnetic field and at remanence, revealing different magnetization states and allowing for step-by-step imaging of magnetization reversal processes.

## Experimental

FEBID was performed in a dual-beam system (FEI Strata DB 235M) using the following electron beam parameters: 1 μs dwell time, 90% overlap and 85 and 130 pA beam currents for energies of 5 and 15 keV, respectively. The base pressure in the chamber was 2.2 × 10^−6^ mbar, whereas during deposition it was 3.3 × 10^−6^ mbar. NWs that had lengths of 2.5–3.0 µm, widths of 70–100 nm and thicknesses in the range of 10–40 nm were deposited directly on C TEM grids. Square rings were formed from 4 NWs of length 1 µm, width 100 nm and nominal thickness 40 nm and were deposited onto both C grids and Si substrates. Their shape was designed using the built-in pattern generator and comprised four rectangles with partially overlapping sides, scanned in parallel. The refresh time between subsequent passes was negligible. This fabrication approach could lead to local small thickness inhomogeneities in areas where the rectangles overlap. The amorphous C and Si substrates were used for TEM and MFM studies, respectively. All depositions and analyses were carried out at room temperature. Although most of the deposited material consisted of Co, some C and O were present in the fabricated structures due to incomplete dissociation of the precursor molecules. In particular, a more significant halo of deposited material was observed near the structures grown on Si. This was caused by larger secondary electron generation by the primary beam interacting with the Si substrate, which is thicker and denser than the C substrate. Energy-dispersive X-ray spectroscopy (EDX) provided measured compositions that depended on the substrate and deposition energy. A spectrum recorded from the bare substrate was subtracted from the sample spectrum in order to remove the substrate contribution. Each concentration measurement was affected by a 5% uncertainty. After subtraction, the overall uncertainty is estimated (from the squared sum of uncertainties of the two spectra) to be 7%. For the samples on C grids, the measured Co concentration was up to (69 ± 5) atom % for 5 keV deposition and (61 ± 4) atom % for 15 keV deposition. For deposition on Si at 5 keV, the Co concentration was measured to be up to (74 ± 4) atom %. Structural information about the NWs was known from a previous study [23]. Briefly, they consist of nanocrystalline Co grains embedded in a carbonaceous matrix. Selected area electron diffraction shows a mixture of hexagonal close-packed (HCP) and face-centered cubic (FCC) Co crystal structures [23].

Magnetic characterization in the TEM was carried out using off-axis EH and L-TEM in Fresnel mode [24]. We used a JEOL JEM-2010 TEM operated at 200 kV for L-TEM and an FEI Titan TEM operated at 300 kV for off-axis electron holography. L-TEM is an imaging technique that enhances local phase gradients, such as those associated with the presence of magnetic domain walls [25–26]. Fresnel images were taken underfocus in low magnification mode, using the objective mini-lens as the imaging lens, with the objective lens slightly excited (at 10% of the value used for eucentric focus), in order to impart a specimen-tilt-angle-dependent magnetic field to the sample. Off-axis electron holography [27] is an interferometric technique that allows the retrieval of real-space phase images of samples in the TEM [28], from which information about the projected electrostatic potential and in-plane magnetic field within and around the specimen can be determined. In the present study, a voltage of 53 V was applied to an electron biprism when recording off-axis electron holograms. Measurements involved acquiring an electron hologram of the sample, a vacuum reference electron hologram and then repeating the measurements with the sample turned over. Reconstruction of the electron holograms was performed using the software Holoworks [29]. The reconstructed phase images were aligned with each other digitally and half of the difference between them was evaluated to subtract the mean inner potential contribution to the phase, in order to obtain the magnetic contribution to the phase alone [30], both outside and inside the sample. MFM is a microscopy technique that is closely related to atomic force microscopy (AFM) [31]. The scanning tip is magnetized and is therefore sensitive to magnetic fields generated by the sample. Attractive and repulsive forces between the tip and the sample are measured and a two-dimensional magnetization map can be recorded. MFM analysis was performed with a VEECO EnviroScope system, working in tapping mode with amplitude detection feedback. The MFM maps were acquired in two-pass lift-mode, with the magnetic signal collected about 30 nm above the surface. The probe used was a Veeco MESP probe, which was made from Sb-doped (n) Si covered by CoCr. The tip was magnetized out-of-plane in a 0.32 T field.

## Results and Discussion

### Electron holography of nanowires

**Figure 1:**
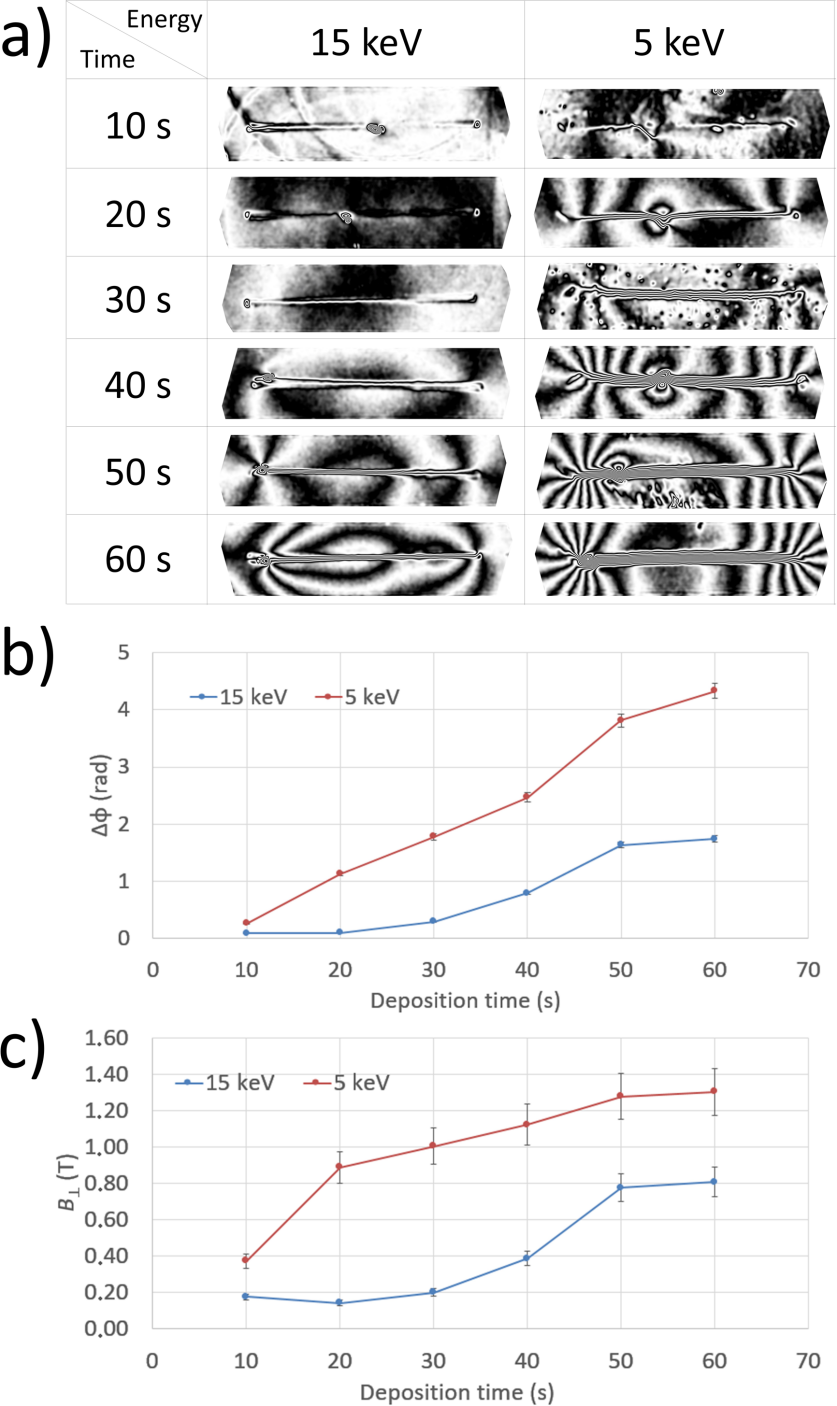
(a) Cosine of 18 times the magnetic phase shift recorded using EH from Co NWs deposited at electron beam energies of 15 and 5 keV for increasing deposition times (from 10 to 60 s). The black equiphase contours have a dipolar form, in particular for longer deposition times and for deposition at 5 keV, leaving one vertex of each NW and entering the opposite one. (b) Steps in phase Δφ across the NWs, measured at their mid-points, as a function of deposition time. 5 keV deposition results in larger values of Δφ than 15 keV deposition. (c) Magnetic induction 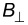 values of the NWs calculated from Δφ, showing saturation for longer deposition times and higher values for the lower deposition energy.

A first magnetic analysis was carried out on the NWs using off-axis electron holography. The measurements were performed on as-deposited samples without applying an external magnetic field. Two different sets of NWs on amorphous C substrates were studied: one deposited at a beam energy of 15 keV and another deposited at a beam energy of 5 keV. In each sample, the deposited shape was the same: 2.5 µm in length, 50 nm in width and with deposition times that varied between 10 and 60 s in 10 s steps. For each deposition time, the deposited thickness was found to be similar in each set, as revealed by energy-filtered TEM (EFTEM) thickness maps [32]. The material deposited at 5 keV had a width that was much larger than that at 15 keV. Figure 1a shows the cosine of 18 times the magnetic contribution to the phase recorded using off-axis electron holography from the different NWs.

The strength of the magnetic signal from each NW can be measured semi-quantitatively by counting the number of black contours across it. The magnetic signal can be seen to increase with deposition time, as more material generates a greater magnetic signal. However, 5 keV deposition results in a higher magnetic phase shift than 15 keV deposition for the same deposition time. The magnetic phase contours suggest visually that the NWs form monodomain states, with their magnetization aligned along their long axes due to shape anisotropy [13]. Impurities are present on some samples (especially for the 20 s and 40 s depositions at 5 keV), resulting in flux-closure domain states, which deform the dipole-like phase structure locally. These impurities are unfortunately an unavoidable by-product of our deposition process, since the electron column is not equipped with fast blanking plates and the beam tends to rest in some position for a while before being blanked.

The step in the magnetic contribution to the phase Δφ_max_ across each NW at the mid-point along its length (i.e., where it is maximum), which is approximately proportional to its magnetization multiplied by the cross-sectional area of the magnetic material within it, is shown in Figure 1b. It increases with deposition time and is greatest for 60 s at 5 keV, where it reaches a value of 4.3 rad.

The measurements confirm that deposition at 5 keV results in a stronger magnetic signal than deposition at 15 keV. This difference results in part from the 5–10% higher relative content of Co for the 5 keV depositions, which was revealed using EDX analysis and is consistent with literature data [33–34]. The slightly higher Co content for depositions at lower energy can be interpreted as a thermal effect resulting from power dissipation of the primary beam closer to the surface [35]. Furthermore, the 5 keV depositions are wider than the 15 keV depositions, resulting in a larger amount of magnetic flux across the corresponding NWs.

Based on the electron holographic measurements, it is possible to estimate the in-plane component of magnetic induction 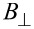 in the sample [36]. For a uniformly magnetized NW, 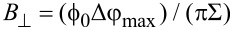, where 
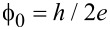
 is the magnetic flux quantum and Σ is the cross-sectional area of the NW. The present NWs are not exactly cylinders: their cross-sectional areas are larger at their bases and their cross-sectional shapes are more Gaussian-like, with some lateral broadening for depositions at 5 keV. Nevertheless, an approximate calculation can be carried out by using the actual cross-sectional areas measured from EFTEM thickness maps. The measured magnetic induction 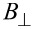 as a function of the deposition time is shown in Figure 1c. For the same deposition time, 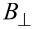 is higher for depositions at 5 keV. There is also an increasing trend with increasing deposition time, which tends to saturate at 50–60 s.

For the 5 keV samples, 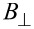 values of up to (1.3 ± 0.2) T are reached for the highest deposition time (60 s). This value is apparently higher than that reported recently [37] for vertical nanopillars with the same Co purity. The discrepancy could be explained by the growth geometry of the deposit, i.e., on-substrate vs vertical. A vertical structure has roughly twice the surface area exposed to oxidation with respect to an on-substrate-grown structure, and the same authors have shown that surface oxidation is responsible for a decrease in magnetic induction by up to 30% in Co nanopillars [38]. Following this argument, the *B* value measured in our nanowires should be 15% higher than that for vertical nanopillars with the same Co purity. This turns out to be in perfect agreement with our recent work [39], where a *B* value of 1.1 T was measured for vertical Co nanopillars having the same composition, within experimental error, as the nanowires presented here. Taken together, these results indicate that on-substrate deposition at lower energy is more suitable for obtaining highly magnetic NWs. The following analyses are therefore presented only for deposition at 5 keV.

### Lorentz TEM of nanowires

Further investigations of the magnetic properties of the NWs and nanorings were carried out using L-TEM. The microscope objective lens was used to apply a vertical magnetic field to the sample, which was tilted to introduce a component of the lens field in the sample plane. The pre-calibrated value of the applied vertical magnetic field *B* was 0.6 T [40]. For a tilt angle θ, the in-plane magnetic field applied to the sample was *B*_eff_ = *B*·sin θ. The angle was varied over a 3.0° range in steps of 0.1°, corresponding to a maximum value for *B*_eff_ of 2 × 10^−2^ T. This value was chosen because, for weak applied magnetic fields, the sample is not at saturation and different magnetic domain configurations can be studied.

**Figure 2:**
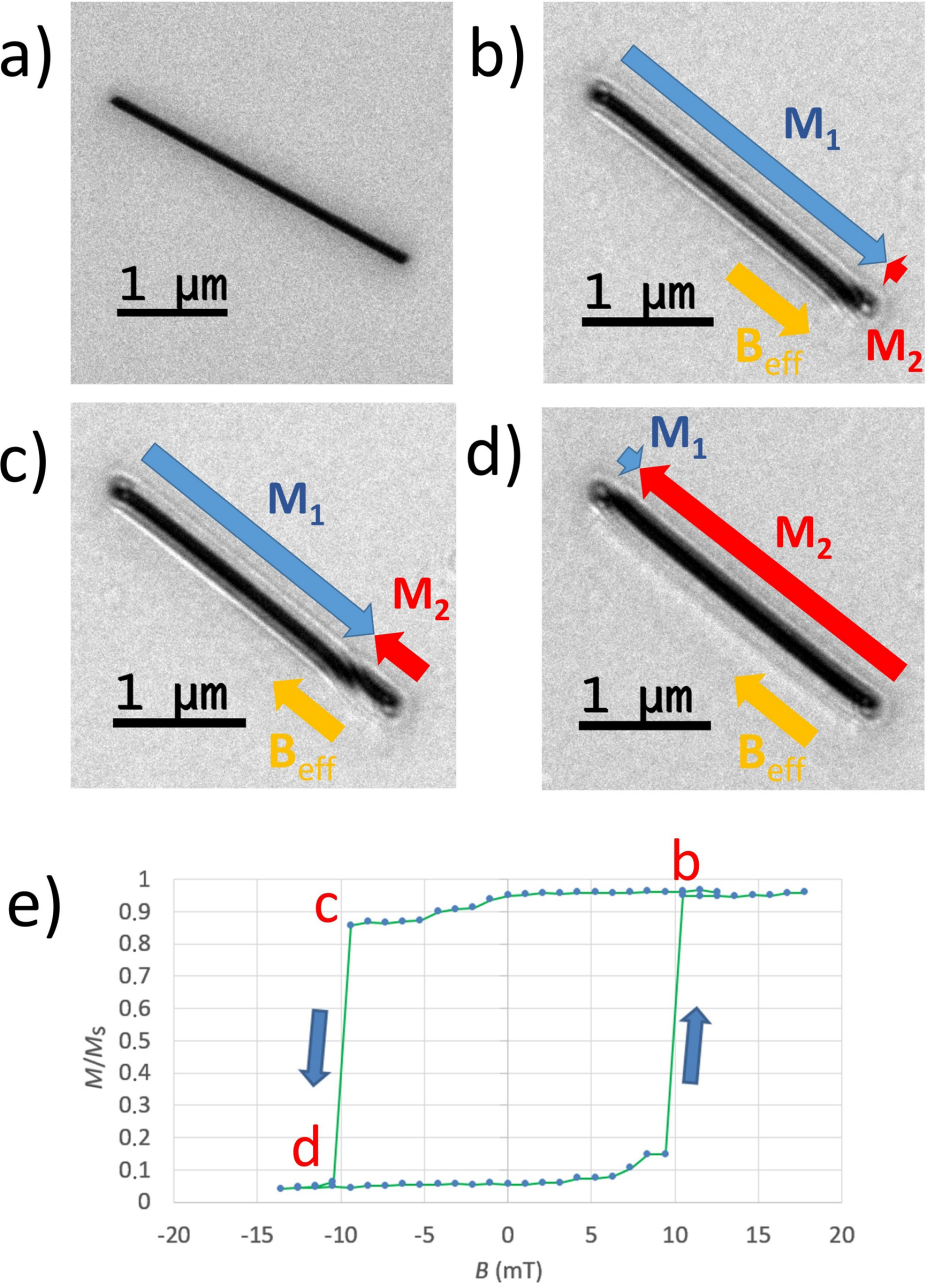
(a) In-focus bright-field TEM image of a Co NW. (b) L-TEM underfocus image of the NW, exhibiting a kink at the right end due to a magnetic domain wall. The kink moves leftwards along the NW in (c) and (d) as the specimen tilt angle is increased, thus increasing the leftward-oriented component of the lens field in the sample plane (*B*_eff_). Blue and red arrows mark the opposite magnetic domains M1 and M2, respectively. (e) Hysteresis loop of the NW measured as a function of the in-plane lens field. The normalized magnetization *M*/*M*_s_ was determined by dividing the blue magnetic domain (M1) length by the total NW length. A saturated NW would have *M*_1_/*M*_s_ = 0, 1. The letters indicate the points corresponding to panels b–d.

A representative image of a NW is shown in Figure 2a for deposition at a beam energy of 5 keV on a 3 × 0.05 µm^2^ area for 60 s, resulting in a length of 3 µm and a width of ca. 100 nm. When observed out of focus using L-TEM, the bright and dark fringes along its sides exhibit an asymmetry that is related directly to its local magnetization direction. When two magnetic domains with opposite directions are present in the same NW, the fringe contrast changes at a domain wall in a manner that can be described as a “kink” [41]. In Figure 2b, the Fresnel fringes have uniform contrast along almost the whole length of the NW, except for a kink at the right end, indicating that the NW has a uniform magnetic domain M1 and a smaller opposite magnetic domain M2. As the sample tilt is increased (Figure 2c), corresponding to an increasing external field *B*_eff_, the domain M2 increases in length at the expense of domain M1 and the kink shifts leftwards. This shift is slow for a wide angular range, until at a value of θ of approximately −1.0° the kink abruptly moves to the left and domain M2 extends over almost the whole length of the NW, as shown in Figure 2d. As all of the images were acquired with the *B* field applied, this can be regarded as a dynamic process. However, the temporal resolution is limited by the tilt steps and the camera acquisition time. All of the domain shifting and reversal processes occur within this limited timescale. Therefore, time-resolved imaging of magnetization switching is unfortunately not possible.

An interesting analysis can be carried out by plotting the ratio of the length of the M1 domain to the length of the NW as a function of *B*_eff_, as shown in Figure 2e. A value of 1 or 0 for the ratio corresponds to the NW adopting single-domain states M1 or M2, respectively. Magnetization saturation (*M*_s_) is then reached and no kink is present. In the plot of the experimental data points shown in Figure 2e, the letters b, c and d correspond to the images shown in Figure 2b, Figure 2c and Figure 2d, respectively. The square shape of the loop is a sign of the single domain character that results from the high aspect ratio and shape anisotropy of the NW. A coercive field of approximately 10 mT is measured from the loop and is consistent with previous measurements on Co NWs [42]. High coercivity is an important property in applications, as it ensures a stable magnetic state in such a nanostructure. The NWs presented here have a small enough diameter to behave in a single-domain-like manner. Higher coercivities could be achieved by depositing NWs with smaller widths.

### Lorentz TEM and MFM of square nanorings

**Figure 3:**
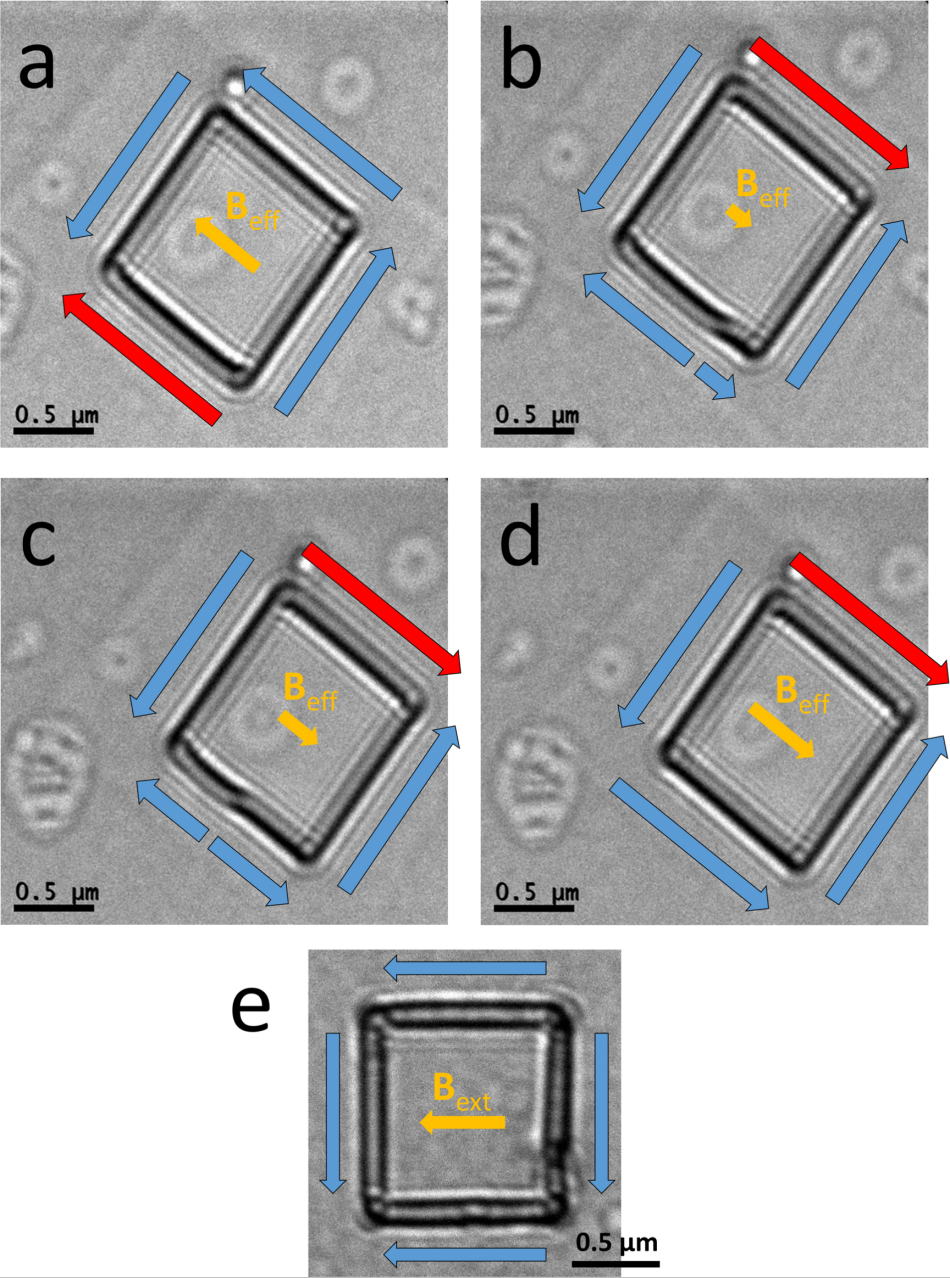
L-TEM images of square rings, showing the magnetization directions under different magnetic fields *B*_eff_ and at remanence after applying an external magnetic field *B*_ext_. The series starts from a saturated horseshoe state in (a). When the external field is reversed, a mixed state is visible, with the lower-left side exhibiting a kink generated by a domain wall (b). As the external field is increased, the domain wall shifts in the direction opposite to the field (c). When *B*_eff_ is increased to its maximum value, the kink vanishes and the horseshoe state appears again with a 180° rotation (d). (e) L-TEM image obtained after applying a higher magnetic field (0.3 T) along the side, before the insertion of the sample. The magnetization state is then observed at remanence, and reveals an onion state.

Two square nanorings, each composed of four NWs, were deposited on amorphous C and studied using L-TEM. The first nanoring was deposited at 5 keV. Each NW was deposited by scanning the beam for 50 s on a 1 × 0.05 μm^2^ area, resulting in sides that are 1 μm long and ca. 100 nm wide. When observed out of focus, as shown in Figure 3a, a value for *B*_eff_ of 4 × 10^−2^ T oriented nearly parallel to one side results in a magnetic domain structure that is referred to as a “horseshoe” state [43]. This arrangement consists of three consecutive domains that make a “horseshoe” shape and a fourth domain, at the bottom left, which is aligned in the opposite way, in response to the direction of the applied magnetic field. In the following, the ability of L-TEM to provide a direct visualization of the switching process of the horseshoe state in a square nanoring is demonstrated. Just as for nanowires, the tilt angle was varied and the square nanoring imaged with various *B*_eff_ values applied during the process. When *B*_eff_ is reversed and then gradually increased up to a value of −0.6 × 10^−2^ T (Figure 3b), the two domains that are perpendicular to the direction of the applied field are not affected. In contrast, the upper right domain reverses immediately, aligning with the field, while the lower left domain undergoes a gradual transition, supporting two opposing domains that are revealed by the presence of a kink. As the field is increased to a value of −1.7 × 10^−2^ T (Figure 3c), the domain wall moves in order to increase the fraction of the NW that is aligned with the field. When the sample is brought towards saturation (Figure 3d), the kink vanishes and a horseshoe state appears again, but with a 180° rotation that brings the domain that is in the opposite direction to the consecutive domains to the upper right. The reversal of upper-right and lower-left sides at different field values is a consequence of their non-identical size, as shown by the micromagnetic calculations in [18]. The circular dot outside the square, near the top corner, is unfortunately one of the unavoidable by-products of our deposition process. This feature does not influence the overall magnetic arrangement of the square.

A second nanoring was deposited with the same parameters, with the aim of studying its remanent magnetization state after the application of higher fields. In the TEM, in fact, the narrow tilting angle range limits the effective field values. For this reason, a nanoring was magnetized outside the microscope using an electromagnet, and applying a field *B*_ext_ of 0.3 T along its side. The resulting L-TEM image, which is shown in Figure 3e, reveals a magnetization state that is different from all those previously observed, and is characterized by two pairs of consecutive domains running along the sides of the square symmetrically with respect to the diagonal. Such a magnetization arrangement is referred to as an “onion” state [43]. By carrying out a quantitative analysis on Figure 3e, a contrast difference of 7–15% is revealed between the inner and outer fringes on each side of the square, which permits to assign a leftward domain orientation of the top and bottom sides and a downward orientation of the left and right sides. In the lower part of the right side a small dot corresponds to an imperfection caused by the limitations of our scanning setup. This flaw is similar to the one previously mentioned, and can be regarded as a small magnetic nanopillar causing a local magnetization rearrangement along the side [44]. This, however, does not seem to perturb the overall magnetic onion state, which is confirmed also by the analyses presented in the next paragraph.

Horseshoe and onion states have been observed during the switching of square permalloy rings, when slight ring asymmetry [43] or slight misalignment of the external field from the square side [17] are present. By decreasing the external field from saturation, when all four domains are aligned with the direction of the applied field, an onion state is first observed, followed by a horseshoe state at a lower field. The present L-TEM results are consistent with this behavior, as the external field applied in the case of the onion state is higher than that applied during horseshoe state observation.

**Figure 4:**
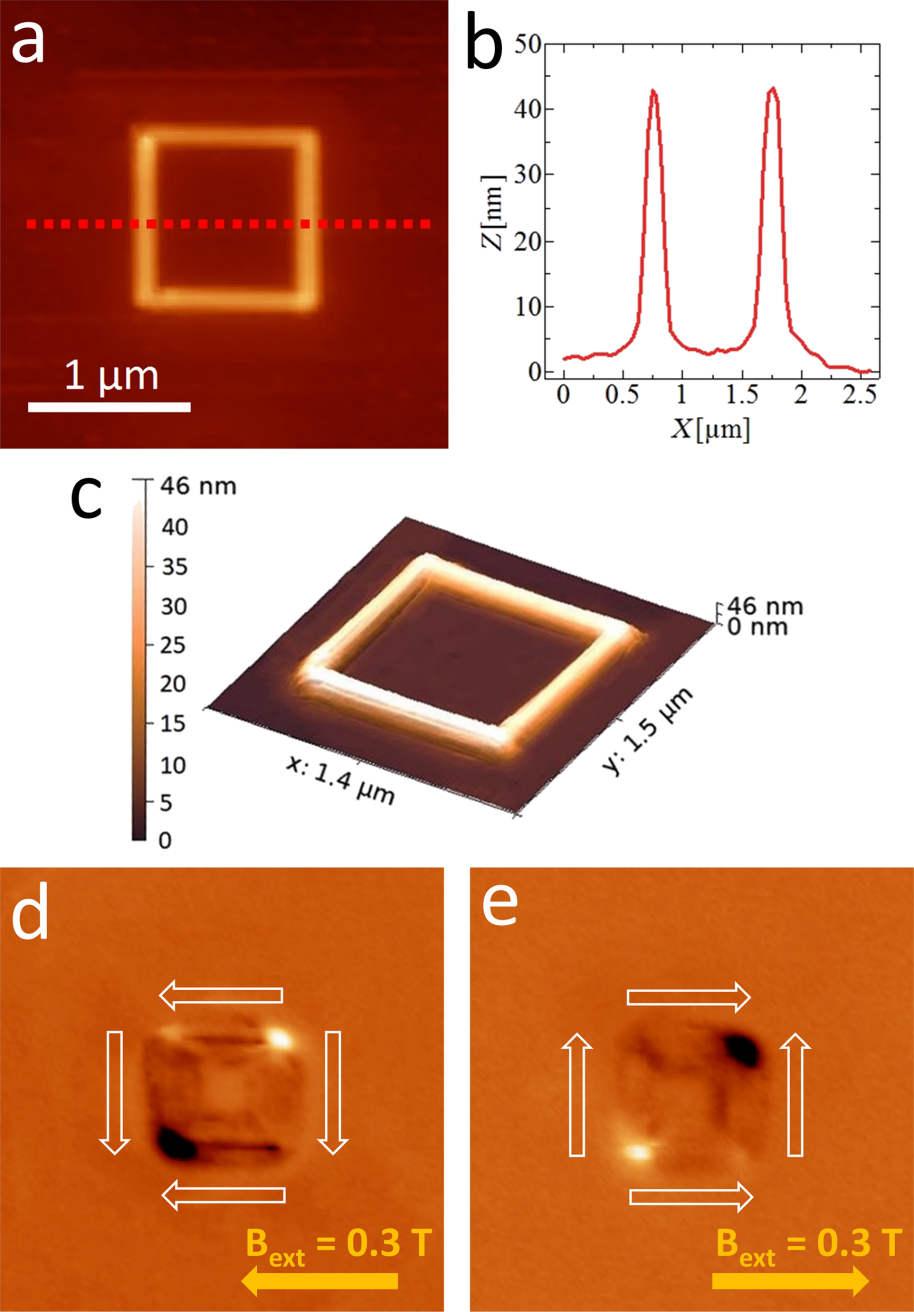
(a) AFM image of a square ring. (b) Height profile corresponding to the red dashed line in (a). (c) 3D AFM topographic map of the area in (a). (d, e) MFM images of the square ring after applying a 0.3 T external field oriented along the side (d) towards the left and (e) towards the right. Dark (bright) areas are representative of attractive (repulsive) magnetic forces between the tip and the sample. This domain arrangement, with bright and dark spots at the vertices of the diagonal, provides evidence for a magnetic onion state, as indicated by the white arrows.

In order to validate the L-TEM results obtained at remanence and to measure the three-dimensional (3D) topography of the sample, additional analyses were carried out using AFM and MFM. A square nanoring was deposited at 5 keV on a Si substrate for MFM analysis. An AFM topography image is shown in Figure 4a, while a height profile taken across the middle of the nanoring is shown in Figure 4b.

Both sides have a thickness of ca. 40 nm, a length of 1 μm and a width of ca. 100 nm. A topographic map of the same area is shown in Figure 4c. In this map, a slight accumulation of deposit at the corner is visible. However, it does not appear to affect the overall magnetic state. Before performing MFM, the same external magnetic field *B*_ext_ of 0.3 T, aligned with one of the sides, was applied outside the microscope. After the first analysis, it was then applied in the opposite direction. As shown in Figure 4d and Figure 4e, the remanent magnetization is aligned with a diagonal of the square and reverses along the same diagonal after applying an opposite external field. The magnetic signal is visible in the form of bright and dark spots at opposite corners of the square, which correspond to repulsive and attractive forces on the tip, respectively. This is another manifestation of an onion state, in agreement with what previously observed using L-TEM. The presence of halo deposition near the square ring contributes to the MFM signal from its surroundings. However, due to the low Co deposit amount in this halo, it does not affect the overall magnetization state of the nanostructure. The halo may at most slightly modify the coercivity of the nanostructures [45].

## Conclusion

Co NWs and square nanorings were deposited using FEBID from a Co carbonyl (Co_2_(CO)_8_) precursor and characterized magnetically using both TEM and MFM. EH measurements on as-deposited NWs revealed single-magnetic-domain states, with a higher magnetic signal for 5 keV deposition than for 15 keV deposition. This difference is thought to result from both a greater relative Co content (by 5–10% for the 5 keV deposition than for the 15 keV deposition) and a difference in NW width. L-TEM analysis of both the NWs and the square rings provided insight into the effect on magnetic domain structure of a weak external magnetic field. L-TEM images of NWs were used to reveal the presence of a square hysteresis loop with a coercive field of approximately 10 mT. L-TEM images of square nanorings revealed a horseshoe magnetic state, which could be changed to an opposite horseshoe state by reversing the magnetic field applied in situ. By increasing the external magnetic field and observing the nanorings at remanence, L-TEM and MFM analyses revealed the formation of a magnetic onion state. Our results confirm that FEBID is a suitable technique for depositing magnetic nanostructures with tailored geometries and that EH, L-TEM and MFM provide complementary information about their static and dynamic magnetic properties.
